# Rapid transcriptome characterization and parsing of sequences in a non-model host-pathogen interaction; pea-*Sclerotinia sclerotiorum*

**DOI:** 10.1186/1471-2164-13-668

**Published:** 2012-11-26

**Authors:** Xiaofeng Zhuang, Kevin E McPhee, Tristan E Coram, Tobin L Peever, Martin I Chilvers

**Affiliations:** 1 Department of Plant, Soil and Microbial Sciences, Michigan State University, 1066 Bogue Street, East Lansing, MI, USA; 2 Department of Plant Sciences, North Dakota State University, 370G Loftsgard Hall, Fargo, ND, USA; 3 Dow AgroSciences LLC, 9330 Zionsville Road, Indianapolis, IN, USA; 4 Department of Plant Pathology, Washington State University, Pullman, WA, USA

**Keywords:** *Pisum sativum*, *Sclerotinia sclerotiorum*, Transcriptome, Parsing of host-pathogen sequences, Non-model organism

## Abstract

**Background:**

White mold, caused by *Sclerotinia sclerotiorum*, is one of the most important diseases of pea (*Pisum sativum* L.), however, little is known about the genetics and biochemistry of this interaction. Identification of genes underlying resistance in the host or pathogenicity and virulence factors in the pathogen will increase our knowledge of the pea-*S*. *sclerotiorum* interaction and facilitate the introgression of new resistance genes into commercial pea varieties. Although the *S*. *sclerotiorum* genome sequence is available, no pea genome is available, due in part to its large genome size (~3500 Mb) and extensive repeated motifs. Here we present an EST data set specific to the interaction between *S*. *sclerotiorum* and pea, and a method to distinguish pathogen and host sequences without a species-specific reference genome.

**Results:**

10,158 contigs were obtained by *de novo* assembly of 128,720 high-quality reads generated by 454 pyrosequencing of the pea-*S*. *sclerotiorum* interactome. A method based on the tBLASTx program was modified to distinguish pea and *S*. *sclerotiorum* ESTs. To test this strategy, a mixture of known ESTs (18,490 pea and 17,198 *S*. *sclerotiorum* ESTs) from public databases were pooled and parsed; the tBLASTx method successfully separated 90.1% of the artificial EST mix with 99.9% accuracy. The tBLASTx method successfully parsed 89.4% of the 454-derived EST contigs, as validated by PCR, into pea (6,299 contigs) and *S*. *sclerotiorum* (2,780 contigs) categories. Two thousand eight hundred and forty pea ESTs and 996 *S*. *sclerotiorum* ESTs were predicted to be expressed specifically during the pea-*S*. *sclerotiorum* interaction as determined by homology search against 81,449 pea ESTs (from flowers, leaves, cotyledons, epi- and hypocotyl, and etiolated and light treated etiolated seedlings) and 57,751 *S*. *sclerotiorum* ESTs (from mycelia at neutral pH, developing apothecia and developing sclerotia). Among those ESTs specifically expressed, 277 (9.8%) pea ESTs were predicted to be involved in plant defense and response to biotic or abiotic stress, and 93 (9.3%) *S*. *sclerotiorum* ESTs were predicted to be involved in pathogenicity/virulence. Additionally, 142 *S*. *sclerotiorum* ESTs were identified as secretory/signal peptides of which only 21 were previously reported.

**Conclusions:**

We present and characterize an EST resource specific to the pea-*S*. *sclerotiorum* interaction. Additionally, the tBLASTx method used to parse *S*. *sclerotiorum* and pea ESTs was demonstrated to be a reliable and accurate method to distinguish ESTs without a reference genome.

## Background

White mold, caused by *Sclerotinia sclerotiorum* (Lib.) de Bary, is a devastating disease of over 400 reported dicotyledonous hosts [1]. The disease causes economically significant losses of many crop plants including pea (*Pisum sativum* L.) under the appropriate environmental conditions [2]. Currently, little is known about the genetic control of pathogenicity in the fungus and mechanisms of resistance in pea. Although hundreds of pea cultivars have been screened for white mold resistance in replicated greenhouse and laboratory tests [3], only partial resistance has been identified to date.

The identification of genes underlying *S*. *sclerotiorum* pathogenicity and resistance in pea would increase our knowledge of the pea-*S*. *sclerotiorum* interaction and facilitate the introgression of resistance into pea varieties. However, progress in these areas has been hampered by the lack of sequence information regarding the pea genome. Although other legume genomes, including the models *Medicago truncatula*, *Lotus japonicus* and economically important *Glycine max* (soybean) are available [4], *Pisum sativum* is still genome resource-poor in part due to the large genome size and large fraction of highly repetitive DNA [5].

The performance of Next-Generation sequencing (NGS) technologies continue to rise while costs continue to fall which enables researchers to conduct whole transcriptome sequencing (RNAseq) studies of interactions between plants and pathogenic fungi [6]. The application of NGS in plant-fungal interaction research promises to shorten the overall time of development of molecular genetic information necessary for functional and translational studies. However, RNAseq has rarely been used to study plant-pathogen interactions, particularly in non-model systems. One reason for this is the difficulty in distinguishing plant and fungal ESTs and even virus or viroid contamination, particularly when reference genomes are not available. Here we report novel transcriptome sequence information from the pea-*S*. *sclerotiorum* interaction obtained by 454 pyrosequencing and propose a method of rapid and efficient transcriptome characterization in a non-model species with little prior molecular information. We also report on the development and validation of a strategy to distinguish plant and fungal ESTs using the tBLASTx program and “proxy-reference” genomes in the absence of true reference genomes.

## Results

### Contiguous EST assembly

**Figure 1:**
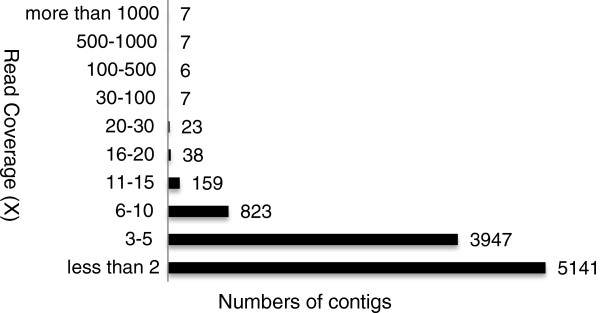
**Depth of 454 pyrosequence read coverage (X) for the 10,158 pea-*S.sclerotiorum *transcriptome EST contigs** .

10,158 contigs were obtained by *de novo* assembly of 128,720 high-quality reads produced on a Roche 454 GS FLX sequencer (see Additional file 1). Minimum contig length was 50 bp, maximum length was 1,015 bp and average length was 200 bp. Average read coverage of contigs was 4.5X, and the maximum read coverage was 2,303X (Figure 1).

### Filter for virus or viroid contamination

The tBLASTx program identified 51 contigs with a BLAST hit (alignment identity) to virus or viroid DNA with an e-value cutoff threshold of less than 1e^-3^. Further evaluation of these 51 EST contigs with tBLASTx against 3 legume and 7 fungal genome databases (which acted as proxy-reference genomes) revealed that 46 contigs showed significant alignment with the proxy plant genome database, 43 showed significant alignment with the fungal genome database and 40 showed significant alignment with both databases, 2 contigs showed significant alignment only with virus genomes. By comparing the e-value ratio (virus/fungi or virus/plant) of all proxy-reference genome alignments, 10 contigs were assigned to pea and 9 contigs were assigned to *S*. *sclerotiorum* based on an e-value ratio >1e^20^, 30 contigs were difficult to distinguish, with e-value ratios between 1e^-20^ and 1e^20^. BLASTn analysis of the 32 unassigned contigs against pea and *S*. *sclerotiorum* ESTs from known sources revealed that 20 contigs, including the 2 that only aligned with virus genomes, had high identity matches to pea with 95% accuracy and 95% query coverage, and 1 to *S*. *sclerotiorum*. The 11 contigs which were difficult to assign to a genome database by either tBLASTx or BLASTn methods were far more similar to plant or fungi than to virus genomes.

### Development and testing a method to distinguish pea and *S*. *sclerotiorum* ESTs using an artificially mixed pool

**Figure 2:**
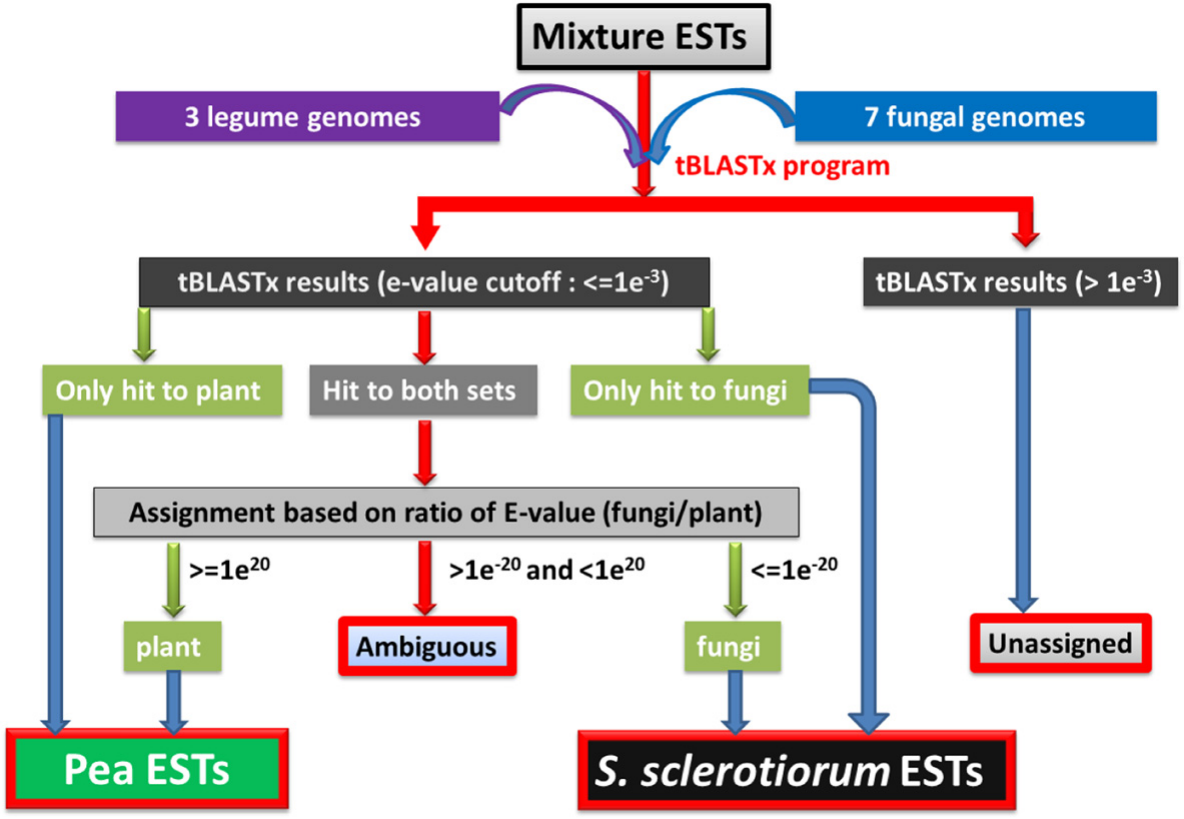
**Flow diagram of host and pathogen EST parsing method for mixed transcriptomes from plant and fungi based on a modified tBLASTx method** .

Pea and *S*. *sclerotiorum* ESTs were downloaded from GenBank to test the tBLASTx sorting method. Three hundred twenty-one ESTs with vector contamination and 71 ESTs highly similar to virus or viroids were removed from the total pool of 36,080 known pea and *S*. *sclerotiorum* ESTs. Using an e-value threshold of 1e^-3^, 35,688 mixed ESTs from pea and *S*. *sclerotiorum* were compared to legume and fungal proxy-reference genome databases and parsed using the tBLASTx program (Figure 2). 11,191 ESTs only aligned with the legume proxy-reference genomes, 11,259 ESTs only aligned with the fungal proxy-reference genomes, 11,266 ESTs similar to both plant and fungal proxy-reference genomes and 1,972 ESTs did not match either proxy-reference genome database. The ESTs with tBLASTx results to both plant and fungal genomes were analyzed further by comparing the e-value ratio (fungi/plant) of fungal and plant proxy-reference genome alignments. 4,098 ESTs were assigned to pea based on an e-value ratio >1e^20^, 5,649 ESTs were assigned to *S*. *sclerotiorum* based on an e-value ratio <1e^-20^, while 1,519 ESTs were difficult to distinguish due to high e-value alignments to both proxy-reference genome databases, with e-value ratios between 1e^-20^ and 1e^20^. This method successfully separated 90.1% of the known ESTs into pea or *S*. *sclerotiorum* categories, with only a 0.1% misallocation rate. Only 5.5% of ESTs had zero similarity to either of the proxy-reference genomes, and 4.3% of ESTs had high similarity to both the plant and fungal proxy-reference genome databases (Table 1).

**Table 1:** **Preliminary testing of the tBLASTx method with pooled known pea and *S*. *sclerotiorum *ESTs**

**Category of EST**	**Numbers of ESTs**	**ESTs assigned successfully**
Plant	15,289	99.9% (14 wrong)
Fungi	16,908	99.8% (23 wrong)
Ambiguous	1,519	N/A
Unassigned	1,972	N/A
**Total**	35,688*	90.1%

*To assess the potential of the tBLASTx method for the parsing of pea and *S*. *sclerotiorum* EST contigs, 17,533 and 18,547 known *S*. *sclerotiorum* and pea ESTs from online databases were pooled, the ESTs were then parsed using the tBLASTx method. Only 9.8% of ESTs could not be assigned and only 37 (0.1%) ESTs were mis-assigned to the wrong class (plant or fungi).

### Parsing 454 pyrosequence pea and *S*. *sclerotiorum* ESTs with tBLASTx and BLASTn

**Figure 3:**
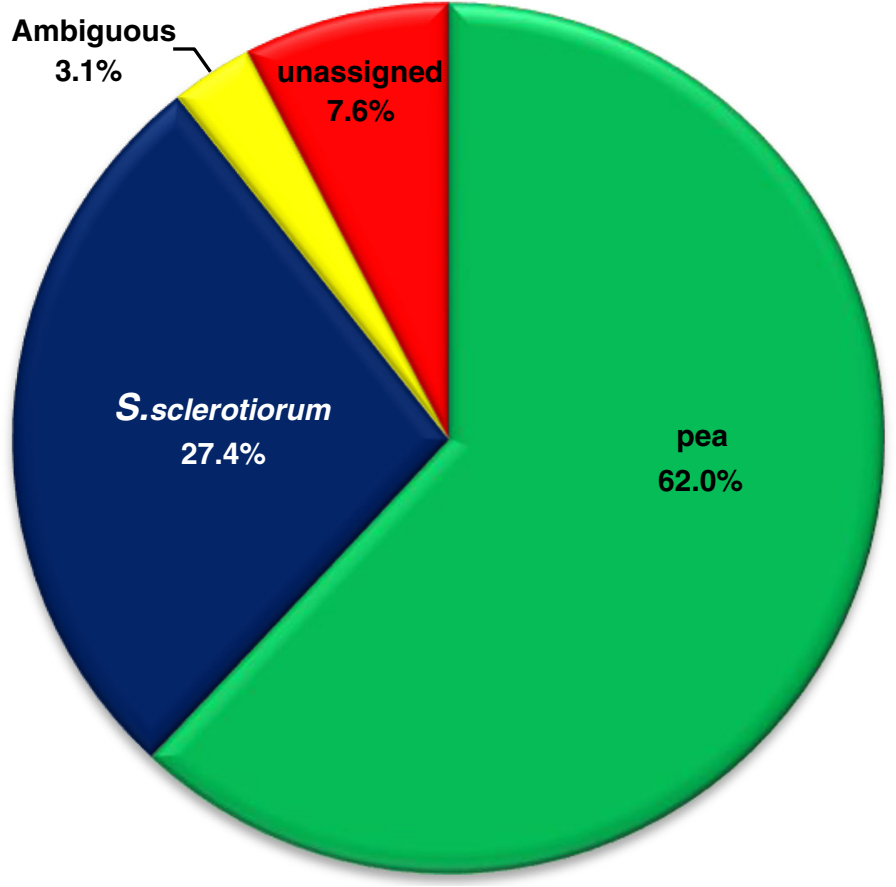
**Distribution and assignment of 10**,**158 EST contigs derived from pyrosequencing of the pea-*Sclerotinia sclerotiorum *transcriptome using the tBLASTx method.** EST contigs with an e-value score <1e^-3^ to only plant or fungal EST reference databases were automatically assigned. Those EST contigs with hits (<1e^-3^) to both databases were assigned based on a cutoff ratio of e-values (fungi/plant), those ESTs with a ratio > =1e^20^ were classified as pea, those < =1e^-20^ as Sclerotinia and those between >1e^-20^ and <1e^20^ as ambiguous. ESTs without any hits (>1e^-3^) were classified as unassigned ESTs.

Initial tBLASTx parsing of 10,158 contigs with an e-value threshold of 1e^-3^ resulted in identification of 4,523 pea ESTs, 2,304 *S*. *sclerotiorum* ESTs, 1,974 ESTs that matched both pea and *S*. *sclerotiorum*, and 1,357 ESTs that did not matched either proxy-reference genome database. The 1,974 ESTs that matched both proxy-reference genomes at the e-value threshold of 1e^-3^ were further subdivided using the e-value ratio method into 544 pea ESTs (fungi/plant e-value ratio >1e^20^), 355 *S*. *sclerotiorum* ESTs (fungi/plant e-value ratio <1e^-20^) and 1,075 that were ambiguous (fungi/plant e-value ratio <1e^20^ and >1e^-20^). This brought the number of classified ESTs for each category to 5,067 for pea, 2,659 for *S*. *sclerotiorum*, 1,075 as ambiguous with high matches to both proxy-reference genomes, and 1,357 with no significant alignment. The remaining 2,432 EST contigs that were ambiguous or showed no significant alignment were further parsed with BLASTn analysis against known pea and *S*. *sclerotiorum* ESTs if identity and query coverage were both equal to or greater than 95%. 1,232 ESTs of this pool were assigned with BLASTn to pea and 121 ESTs were assigned to *S*. *sclerotiorum*, leaving 310 ambiguous and 769 EST contigs with no significant alignment. In total with tBLASTx and BLASTn, 10,158 contigs were separated into 6,299 pea ESTs, 2,780 *S*. *sclerotiorum* ESTs, 310 ambiguous ESTs and 769 unassigned ESTs (Figure 3).

### Validation of tBLASTx and BLASTn EST parsing results by PCR

**Figure 4:**
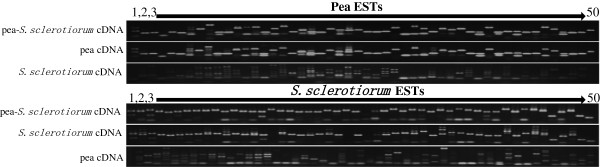
**PCR results for validation of 50 pea and 50** ***S.sclerotiorum *pyrosequence-classified EST contigs against a mix of host and pathogen interaction cDNA, host cDNA only and pathogen cDNA only.** All 50 PCR pea primer sets amplified the expected amplicons from the pea-*S*. *sclerotiorum* and pea cDNA samples. Forty-seven of the 50 PCR *S*. *sclerotiorum* primer sets amplified expected amplicons from the pea-*S*. *sclerotiorum* and *S*. *sclerotiorum* cDNA samples, one primer pair failed to amplify from either cDNA and the remaining two only amplified product from the *pea*-*S*. *sclerotiorum* cDNA possibly indicating interaction induced transcripts.

Validation of the tBLASTx and BLASTn assignment was performed for 50 *S*. *sclerotiorum* and 50 pea EST contigs randomly sampled from the two assigned categories. All 50 primer sets designed to the pea EST contigs amplified the expected amplicon size in both the pea-*S*. *sclerotiorum* and non-inoculated pea cDNA indicating correct parsing assignment of the pea ESTs (Figure 4). Of the 50 PCR primers designed to the *S*. *sclerotiorum* ESTs, 47 amplified a PCR product from both the pea-*S*. *sclerotiorum* and *S*. *sclerotiorum* only cDNA samples and most of them amplified the same size amplicon in both cDNA samples. Two of the 50 *S*. *sclerotiorum* PCR primer pairs amplified the expected PCR products from the pea-*S*. *sclerotiorum* cDNA sample but not the *S*. *sclerotiorum* only cDNA, perhaps indicating that this transcript is only expressed during the interaction with pea. One *S*. *sclerotiorum* primer set failed to amplify any PCR product from either template.

### Unique ESTs expressed in the pea-*S*. *sclerotiorum* interaction

To detect unique genes expressed in our pea-*S*. *sclerotiorum* interaction, the 6,299 classified pea ESTs in our data set were compared with BLASTn against 81,449 recently published pea ESTs from flowers, leaves, cotyledons, epi- and hypocotyl, and etiolated and light treated etiolated seedlings [7]. Of these 6,299 ESTs, 3,459 ESTs had significant alignments with an e-value cutoff of 1e^-10^, in which 1,668 contigs had a percentage identity threshold of 95% for 95% or more of the query sequence, leaving 2,840 potentially unique pea ESTs to the pea-*S*. *sclerotiorum* interaction. It was possible to annotate 1,631 of these ESTs of which 67 contigs encode transcription factors (Table 2), 69 were involved in signaling pathways (Table 3) and 82 contigs were involved in encoding defense-associated proteins (Table 4).

**Table 2:** Description of genes encoded by 67 unique pea ESTs encoding transcription factors

**Seq.Name**	**Seq**. **Description**	**Seq.Length**	**min.e-value**	**mean Similarity**
MYB transcription factors				
746	myb-like protein	102	7.8E-12	92.1%
914	myb-cc transcription factor	143	7.0E-18	94.8%
1535	myb transcription factor myb50	120	1.5E-07	81.3%
3483	myb-cc transcription factor	264	1.00E-32	84.80%
5012	myb family transcription factor	226	5.90E-20	68.90%
5326	myb family transcription factor	209	3.50E-17	80.70%
5825	myb-related transcription factor	368	8.50E-32	65.60%
AP2/EREBPs transcription factors				
1311	ethylene insensitive transcription factor	220	1.11E-34	79.50%
2887	dehydration responsive element-binding protein 3 (DREB3)	149	6.8E-08	73.5%
4803	ap2 domain-containing transcription factor	150	1.5E-07	66.1%
7472	ap2 erf domain-containing transcription factor	376	7.3E-23	81.30%
7671	ein3-like protein	150	1.1E-13	88.2%
8261	ethylene insensitive transcription factor	163	1.43E-18	90.00%
8894	ap2-like ethylene-responsive transcription factor	228	2.2E-27	78.60%
9486	ap2 erf domain-containing transcription factor	282	5.4E-13	57.80%
WRKY transcription factors				
413	wrky transcription	166	2.4E-18	83.90%
6039	wrky transcription factor 2	277	1.2E-04	47.00%
9142	wrky 10	116	2.2E-14	82.10%
9626	wrky transcription factor 40	124	3.4E-12	85.90%
bZIP transcription factors				
1563	bZIP transcription factor bZIP122	236	2E-28	64.2%
5518	bZip transcription factor	120	1.9E-07	77.7%
Others				
177	transcription factor	196	6.7E-08	98.5%
200	zinc finger family protein	264	3.3E-23	67.4%
257	transcription factor iiia	255	4.8E-30	76.6%
446	histone acetyltransferase	123	1.3E-11	76.6%
642	kruppel-like zinc finger protein	276	3.0E-21	80.1%
677	integral membrane family protein	186	2.4E-21	86.9%
918	ring finger and chy zinc finger domain-containing protein 1	305	1.7E-43	78.3%
1245	zinc finger	178	5.0E-24	72.9%
1407	zinc finger	546	5.7E-60	72.5%
1541	phosphoprotein phosphatase	271	2.3E-45	99.2%
1818	nuclear transcription x-box	246	6.7E-24	77.3%
2070	25.7 kda protein	185	3.5E-12	67.8%
2739	DNA binding protein	254	9.7E-23	80.9%
2864	zinc finger protein 622	156	5.3E-13	88.0%
2982	homeodomain transcription factor	113	2.0E-07	79.7%
3354	dna binding	179	5.5E-15	75.4%
3477	prefoldin subunit 2	218	3.1E-18	87.8%
3616	constans-like b-box zinc finger protein	140	1.7E-11	78.2%
3637	global transcription factor group	108	3.1E-05	80.0%
3724	zinc finger protein	566	1.6E-47	65.2%
3916	knotted I class homeodomain protein	173	7.3E-15	89.7%
4012	ring zinc finger ankyrin protein	141	5.5E-15	87.6%
4123	zinc finger family protein	189	2.0E-28	87.2%
4349	zinc finger ccch domain-containing protein 32	133	2.3E-16	75.2%
4390	C2H2 type zinc finger family protein	232	2.4E-26	81.8%
4632	transcription factor	205	2.7E-17	68.3%
4694	pinoresinol-lariciresinol reductase	347	2.4E-53	92.6%
5015	rna recognition motif and cchc-type zinc finger domain-containing protein	185	1.8E-21	95.4%
5038	transcription factor	198	7.6E-12	82.8%
5161	stress responsive gene 6 srg6	169	5.4E-10	89.8%
5445	ring zinc finger protein	105	2.5E-10	78.4%
6289	transcription factor	247	1.7E-16	88.0%
6595	transcription initiation factor tfiid subunit d5	172	1.4E-13	81.6%
6596	zinc finger ccch domain-containing protein 53	267	4.2E-18	70.1%
6877	transcription factor iiia	217	5.7E-20	73.4%
7318	zinc finger family protein	179	4.7E-14	67.2%
7691	dna binding	167	9.3E-10	69.6%
7815	ring zinc finger protein	154	3.2E-10	71.0%
8103	homeobox protein knotted-1-like 6	183	1.8E-24	86.6%
8133	ribulose bisphosphate carboxylase activase	215	9.1E-34	96.8%
8849	zinc finger (b-box type) family protein	119	4.1E-13	89.4%
9097	zinc finger ccch domain-containing protein 67	225	8.8E-08	62.4%
9224	transcription factor	205	6.8E-05	58.0%
9432	zinc finger	241	3.3E-31	61.4%
9754	Peptide transporter	124	4.2E-10	81.2%
10046	mads-box protein	247	1.8E-26	81.9%

**Table 3:** Description of genes encoded by 69 unique pea ESTs involved in the signaling pathways

**Seq.Name**	**Seq.Description**	**Seq.Length**	**min.e-value**	**mean Similarity**
Abscisic acid mediated signaling pathway				
2884	abscisic acid receptor pyl8	137	2.3E-16	91.5%
3332	serine threonine-protein kinase	116	9.0E-13	89.3%
3469	calcium-dependent protein	190	1.2E-28	96.2%
Auxin mediated signaling pathway				
109	saur family protein	287	3.8E-27	69.3%
1547	auxin-binding protein	247	9.4E-39	87.3%
1737	auxin signaling f-box 3	109	2.2E-11	99.1%
2013	auxin-induced protein	229	2.9E-11	61.8%
7004	germin-like protein	245	5.9E-33	88.4%
7084	auxin down-regulated-like protein	343	4.4E-15	86.9%
8570	auxin-induced protein	177	5.7E-20	80.6%
9301	auxin-induced protein	279	7.4E-44	93.9%
Brassinosteroid mediated signaling pathway				
104	glutamate binding protein	190	9.7E-15	89.7%
516	kinase family protein	250	5.0E-40	97.2%
908	vf14-3-3c protein	290	1.7E-43	92.1%
3415	14-3-3-like protein gf14 lambda	105	3.2E-10	91.5%
8120	kinase family protein	103	1.5E-10	96.5%
9939	BRASSINOSTEROID INSENSITIVE 1-associated receptor kinase 1 precursor	451	5.5E-63	79.2%
Calcium-mediated signaling				
2711	calcium ion binding protein	359	8.9E-21	93.3%
5090	calcium-dependent protein kinase	236	1.2E-36	94.9%
5443	calcium-dependent protein kinase 19	111	2.6E-12	98.2%
5757	calcium ion binding protein	309	3.9E-45	86.2%
8209	calcium-dependent protein kinase 15	123	2.5E-15	85.00%
8909	plasma membrane-type calcium atpase	247	1.5E-36	91.3%
9449	calcium calmodulin-regulated receptor-like kinase	158	7.4E-15	81.4%
10142	calmodulin	365	3.1E-50	100%
Ethylene mediated signaling pathway				
1250	ethylene receptor	232	4.0E-37	73.2%
8377	zinc finger protein, putative	185	1.2E-17	78.6%
Gibberellic acid mediated signaling pathway				
3093	gibberellin oxidase-like protein	205	1.4E-13	78.6%
7394	TPA: putative GID1-like gibberellin receptor	141	4.4E-20	90.05%
7551	gibberellin 2-oxidase	183	4.5E-28	83.0%
10003	gibberellin receptor	259	1.6E-33	75.0%
small GTPase mediated signal transduction				
895	ras-related protein	421	3.9E-48	88.2%
2498	gtp binding protein	159	1.4E-10	96.7%
3413	adp-ribosylation factor	372	7.3E-55	100%
3434	ras-related protein rab7	130	8.6E-16	99.9%
3696	adp-ribosylation factor	140	1.4E-18	99.9%
4176	RAS-related GTP-binding protein	224	9.0E-37	99.1%
4656	small gtp-binding protein	178	3.2E-15	87.4%
5075	small gtp-binding protein	193	3.8E-27	99.0%
5134	gtp-binding protein	282	4.1E-37	95.4%
5318	adp-ribosylation factor	231	1.1E-18	99.9%
7015	adp-ribosylation factor 3	141	1.2E-17	100%
7249	ras-like protein	199	2.1E-30	99.7%
7488	rab2 -family small gtpase	142	9.7E-20	99.7%
8652	gtp binding protein	134	3.9E-16	95.0%
Jasmonic acid or Salicylic acid mediated signaling pathway				
2086	wus-interacting protein 1	236	1.3E-32	91.9%
9051	syntaxin 121	268	2.4E-21	90.3%
9545	bifunctional inhibitor lipid-transfer protein seed storage 2 s albumin-like protein	303	7.0E-34	64.4%
Others				
376	senescence-associated-like protein	194	2.8E-30	93.9%
1216	diacylglycerol kinase 5	156	3.0E-16	82.3%
1266	26 s proteasome non-atpase regulatory subunit 14	158	1.3E-24	99.3%
1756	typical p-type r2r3 myb protein	140	1.8E-05	66.5%
1815	pas lov protein 1	102	1.7E-06	88.0%
2847	peptidyl-prolyl cis-trans isomerase cyp20-3	155	1.3E-22	93.9%
2964	flavin-containing monooxygenase 1	227	2.7E-25	79.6%
4308	small nuclear	231	1.6E-25	94.9%
4474	b regulatory subunit of pp2a ( gamma)	247	7.2E-39	97.8%
5301	actin depolymerizing factor 1	117	5.8E-12	99.6%
5774	atp binding	212	3.5E-25	86.8%
5809	autoinhibited calcium atpase	246	1.2E-04	65.0%
6377	integrin-linked protein kinase family protein	106	3.2E-10	93.6%
6424	protein phosphatase	243	3.1E-34	91.4%
7633	leucine-rich repeat-containing protein	149	2.9E-19	96.4%
8149	dormancy auxin associated protein	193	2.6E-28	80.3%
8514	big map kinase	235	4.4E-28	88.9%
8781	Mitogen-activated protein kinase homolog MMK2	246	1.6E-30	91.3%
9004	protein ralf-like 33	270	3.7E-43	86.4%
9508	ring finger	248	3.2E-31	86.2%
9933	histidine-containing phosphotransfer	221	4.8E-06	75.4%

**Table 4:** Description of 82 unique pea ESTs encoding defense-associated proteins

**Seq.Name**	**Seq.Description**	**Seq.Length**	**min.e-value**	**mean Similarity**
Pathogenesis-related protein				
5422	pathogenesis-related protein	224	5.3E-21	83.9%
6766	pathogenesis-related protein 1	201	8.1E-14	73.1%
7235	pathogenesis-related protein 4a	139	3.2E-07	97.0%
9781	pathogenesis-related protein 1	647	6.3E-58	82.3%
Genes involved in disassembly of fungal cell wall				
196	endo-beta-1,3-glucanase	319	8.7E-48	82.8%
491	chitinase	117	3.4E-12	79.4%
589	chitinase	646	1.5E-96	85.8%
1243	beta-1,3-glucanase	638	1.3E-84	88.0%
1317	transferring glycosyl	257	5.5E-34	77.4%
1622	basic chitinase class 3	451	4.8E-75	84.2%
1687	glycosyl transferase family 8	135	4.7E-14	87.2%
2311	chitinase	184	6.7E-16	79.9%
2469	glycosyltransferase-like protein	225	3.0E-32	85.3%
2880	glycosyl hydrolases family 17 domain-containing protein	206	7.8E-25	84.8%
3896	Glycoside hydrolase, family 17	231	6.4E-19	86.7%
3940	glycosyltransferase family protein	160	8.7E-16	80.7%
4388	beta-1,3-glucanase	243	1.5E-28	85.8%
4424	acidic glucanase	316	4.2E-50	91.3%
5082	aspartyl protease family protein	163	3.2E-10	87.0%
5129	udp-glycosyltransferase-like protein	122	3.1E-13	92.1%
5742	chitinase	123	2.6E-12	86.0%
5908	aspartyl protease family protein	303	2.2E-25	81.0%
6193	acidic glucanase	102	2.7E-12	100.0%
6919	glycoside hydrolase family 47 protein	254	4.5E-12	93.0%
7308	transferase, transferring glycosyl groups	148	2.7E-17	80.9%
8804	glycosyl hydrolase family 81 protein	201	3.7E-19	76.6%
9211	aspartyl protease-like protein	337	8.6E-08	68.7%
Genes involved in biosynthesis of plant cell wall structure				
1677	neutral alpha-glucosidase ab precursor	361	1.6E-38	76.9%
2757	protein cobra	228	4.6E-26	88.2%
4004	cellulose synthase	301	1.7E-11	66.5%
8822	dolichyl-diphosphooligosaccharide--protein glycosyltransferase 48 kda subunit precursor	144	1.8E-16	92.5%
Lignin related genes				
1432	o-methyltransferase 1	525	4.4E-72	79.0%
4536	laccase 8	447	2.4E-82	74.5%
5158	lignin biosynthetic peroxidase	106	1.8E-08	81.3%
5257	caffeic acid 5-hydroxyferulic acid 35-o-methyltransferase ferulic acid complex chain a	171	2.0E-12	83.3%
6534	putative copper ion-binding laccase	208	7.7E-33	77.6%
8248	caffeic acid 5-hydroxyferulic acid 35-o-methyltransferase ferulic acid complex chain a	256	1.9E-26	84.5%
8686	lignin biosynthetic peroxidase	245	7.5E-28	74.8%
Pectin related genes				
150	pectin methylesterase	242	4.1E-26	75.5%
531	21 kda protein	158	1.2E-20	75.9%
892	pectin methylesterase	152	9.1E-21	93.6%
2699	multicopper oxidase	259	1.8E-32	83.6%
7076	pectin methyltransferase qua2	234	1.3E-24	87.9%
8878	pectin lyase-like protein	126	9.2E-10	86.2%
9386	pectin methylesterase	220	2.6E-36	86.3%
Others				
79	threonyl-trna synthetase	184	3.7E-22	89.9%
643	xyloglucan endotransglycosylase hydrolase	215	4.3E-36	97.0%
1215	prolyl 4-hydroxylase alpha	176	7.0E-26	94.5%
1353	(iso)flavonoid glycosyltransferase	240	1.5E-36	85.5%
1357	peroxidase 52	109	2.2E-11	90.8%
1527	60S ribosomal protein L10	530	7.7E-93	97.0%
1813	vacuolar atp synthase subunit	552	1.8E-80	87.2%
2018	gamma-glutamylcysteine synthetase	286	2.4E-37	95.9%
2083	polygalacturonase-inhibiting protein precursor	523	3.0E-89	84.7%
2201	xyloglucan endotransglucosylase hydrolase-like protein	178	1.4E-26	94.1%
2261	3,5-epimerase/4-reductase	199	2.4E-26	92.5%
2418	udp-glucosyl transferase 74b1	224	1.7E-19	79.8%
2760	peptide deformylase 1a	199	6.4E-27	88.6%
2779	alpha-galactosidase 1	296	1.6E-30	77.3%
3004	protein disulfide	245	8.0E-30	92.5%
3304	dolichyl-diphosphooligosaccharide--protein glycosyltransferase subunit dad1	109	1.4E-13	99.6%
3349	pentose-5-phosphate 3-epimerase	171	5.0E-24	95.8%
3877	AKIN gamma	392	1.5E-57	88.0%
4111	polygalacturonase	117	3.4E-12	95.0%
4189	family 8 glycosyl transferase	233	2.1E-17	86.5%
4244	beta-galactosidase	145	1.3E-19	90.9%
4473	nad-dependent epimerase dehydratase	164	9.9E-20	88.6%
4711	udp-d-glucose udp-d-galactose 4-epimerase 2	209	2.6E-28	90.9%
4836	alpha-expansin 4	227	1.4E-37	90.4%
4942	flavonoid glycosyltransferase	368	1.7E-40	73.1%
5625	prolyl 4-hydroxylase alpha	117	4.9E-11	75.7%
5777	nad-dependent epimerase dehydratase	131	1.1E-05	94.0%
6800	putative beta-D-xylosidase	205	1.4E-18	66.9%
6916	UDP-glucose 4-epimerase, putative	322	2.0E-20	88.4%
7235	af137351_1pathogenesis-related protein 4a	139	3.2E-07	97.0%
7414	glycine-rich protein	109	4.5E-12	96.0%
7526	NAD dependent epimerase/dehydratase, putative	273	1.3E-43	97.1%
7528	polygalacturonase precursor	229	2.5E-10	62.0%
8194	protein disulfide isomerase	179	5.0E-24	92.9%
8631	beta-galactosidase like protein	198	1.7E-16	92.3%
8646	nad-dependent epimerase dehydratase	230	2.4E-29	74.1%
8682	polygalacturonase inhibitor protein	582	5.0E-71	80.3%
8736	alanine-2-oxoglutarate aminotransferase 2	221	6.9E-29	87.9%
9036	alcohol dehydrogenase, putative	330	1.3E-35	84.6%
9052	nad-dependent epimerase dehydratase	202	5.6E-23	81.5%

The 2,780 *S*. *sclerotiorum* EST contigs were also assessed with BLASTn against 57,751 *S*. *sclerotiorum* ESTs (from mycelia at neutral pH, developing apothecia and developing sclerotia). Of these, 1,784 ESTs matched with an e-value cutoff of 1e^-10^, in which 294 ESTs matched with 95% identity for 95% of more of the query length to the *S*. *sclerotiorum* EST growth libraries. Of the remaining 996 unique ESTs, it was possible to annotate 438 ESTs of which 95 ESTs were described as being related to pathogen virulence or pathogenicity (Table 5).

**Table 5:** **Description of 95 unique *S*. *sclerotiorum *EST contigs with putative involvement in virulence or pathogenicity**

**Seq.Name**	**Seq.Description**	**Seq.Length**	**min.e-value**	**mean Similarity**
4 genes involved in the recognition of the host and in signaling pathway				
1457	c2h2 type zinc finger containing protein	222	2.2E-35	75.5%
1796	guanine nucleotide-binding protein alpha subunit	171	2.7E-17	95.0%
3623	importin beta-2	109	1.2E-09	85.7%
6785	c2h2 transcription factor	135	2.8E-19	79.9%
18 genes affecting biosynthesis and the integrin of fungal cell walls				
330	alpha-1,2-mannosyltransferase kre5	196	8.4E-19	70.9%
2296	cell wall biogenesis protein glutathione transferase	122	2.8E-14	90.5%
2424	endoglucanase-4 precursor	326	6.4E-27	76.0%
2930	alpha-1,2-mannosyltransferase alg11	154	1.7E-22	95.3%
3362	alpha-glucan-branching enzyme	234	6.8E-29	82.6%
3825	alpha-mannosyltransferase	248	3.4E-41	71.1%
4843	dolichol-phosphate mannosyltransferase	253	1.9E-34	87.1%
5737	endoglucanase ii	122	8.7E-16	86.0%
5773	dolichol-phosphate mannosyltransferase	255	1.9E-26	89.7%
6654	endochitinase 42	189	1.5E-28	87.0%
6797	chitinase	105	2.3E-11	79.9%
6956	endoglucanase ii	267	6.8E-45	86.7%
7397	gpi-anchored cell wall beta-1,3-endoglucanase	159	3.0E-24	69.3%
8474	af215732_1class iv chitin synthase	113	2.0E-07	86.0%
8681	chitin synthase	138	2.0E-20	95.6%
8785	mannan polymerase ii complex anp1 subunit [61]	173	8.6E-24	92.8%
9176	gpi-anchored cell wall beta-1,3-endoglucanase	295	9.2E-34	66.5%
9570	mannosyl transferase	122	3.3E-15	82.6%
12 genes involved in the production of infection structures				
97	siderophore biosynthesis	106	5.9E-12	81.5%
239	mannosyl transferase	221	1.3E-22	94.3%
253	adenylate kinase	140	6.3E-19	88.3%
427	serine threonine-protein phosphatase pp2a catalytic subunit	289	4.9E-51	99.4%
1554	adenylate cyclase	135	1.4E-18	83.2%
4181	mfs toxin efflux pump	194	1.1E-21	69.4%
5419	alcohol dehydrogenase	190	3.4E-28	75.7%
5981	adp-ribosylation factor 1	455	7.3E-23	93.7%
6192	adp-ribosylation factor 1	103	3.5E-12	100.0%
6325	nadp-dependent alcohol dehydrogenase	347	4.0E-53	82.8%
7407	1-phosphatidylinositol phosphodiesterase	102	9.2E-05	76.0%
9579	alcohol oxidase	112	9.7E-15	90.7%
39 genes involved in the penetration of the cuticle and cell wall				
47	cellulose 1,4-beta-cellobiosidase	165	7.3E-23	77.1%
473	xyloglucan-specific endo-beta-1,4-glucanase precursor	228	7.6E-36	84.0%
485	cutinase	375	2.5E-63	68.5%
555	endo-1,4-beta-xylanase	265	3.8E-19	79.3%
717	endo-1,4-beta-xylanase	348	2.8E-62	78.4%
970	acetyl xylan	118	2.3E-16	73.5%
1533	endopolygalacturonase 2	181	1.3E-27	89.5%
1562	carbohydrate esterase family 1 and carbohydrate-binding module family 1 protein	241	4.4E-28	76.1%
2427	4-coumarate:CoA ligase	185	1.8E-29	79.8%
2450	acetolactate synthase	236	1.9E-39	93.3%
3006	aspartic-type endopeptidase	171	1.5E-23	71.2%
3115	fungal alpha-l-arabinofuranosidase	110	3.1E-13	91.6%
3147	extracellular exo-polygalacturonase	269	1.5E-12	83.5%
3513	beta-xylosidase	219	9.1E-37	84.6%
3712	feruloyl esterase b	187	1.7E-27	78.6%
3861	pectin methylesterase	170	2.4E-26	80.0%
3907	extracellular endo-1,5-alpha-l-arabinosidase A	172	1.6E-25	80.0%
4037	aspartic endopeptidase	129	3.2E-18	82.4%
4295	cutinase	278	2.6E-36	66.3%
4446	xyloglucan-specific endo-beta- -glucanase precurso	626	8.3E-89	77.3%
5554	beta-glucosidase 1b	267	8.0E-46	83.7%
5562	alpha-l-arabinofuranosidase	130	1.1E-15	74.2%
5679	polygalacturonase 1	427	9.8E-60	96.6%
5918	acetyl xylan esterase	345	5.1E-16	84.0%
6412	exoglucanase 2 precursor	141	4.8E-19	90.3%
6383	alpha-L-arabinofuranosidase A	130	1.1E-13	78.3%
6431	exopolygalacturonase	135	1.3E-16	89.0%
6759	endo-1,4-beta-xylanase	359	8.8E-45	81.1%
6824	pectin methylesterase	107	3.1E-13	79.7%
7085	pectinesterase family protein	109	7.7E-12	82.5%
7661	polygalacturonase 1	601	3.9E-45	93.2%
7834	extracellular exo-polygalacturonase	386	3.0E-61	68.0%
8501	pectin methylesterase	252	1.6E-41	62.5%
9093	carbohydrate esterase family 8 protein	244	9.4E-31	71.6%
9509	extracellular endo-1,5-alpha-l-arabinase	322	2.0E-36	74.7%
9525	cellulase family protein	239	1.8E-37	72.8%
9583	acetolactate synthase	123	1.3E-16	90.6%
9655	acetyl xylan esterase	129	2.3E-16	82.8%
10069	endopolygalacturonase 2	460	1.2E-73	91.7%
9 genes involved in responding to host immune system				
1220	efflux transporter	120	1.9E-15	80.3%
4783	abc bile acid transporter	142	1.6E-17	72.5%
5659	mfs transporter	236	8.0E-38	80.8%
6180	ATP-binding cassette transporter	229	1.7E-40	81.1%
6538	glutathione transferase	486	3.7E-91	70.2%
7926	glutathione s-transferase ure2-like protein	123	1.3E-16	79.8%
8029	ornithine decarboxylase	234	9.5E-39	90.3%
8188	glutathione s-transferase	527	6.5E-100	79.7%
9716	salicylate hydroxylase	202	5.4E-34	85.8%
3 genes involved in fungal nutrition (virulence-associated)				
1446	methionine aminopeptidase 2	337	7.2E-47	84.8%
1889	vacuolar atpase proteolipid subunit c	314	2.6E-23	92.5%
5131	vacuolar atpase proteolipid subunit c	157	3.1E-21	90.4%
Others 10 genes related with pathogen virulence or pathogenicity				
145	acid proteinase	583	1.8E-89	63.5%
1387	methylcitrate synthase precursor	182	1.7E-27	96.2%
2840	centromere kinetochore protein	217	4.1E-34	73.0%
3499	vacuolar protein 8	255	7.7E-28	97.4%
3747	acid proteinase	246	6.5E-40	66.9%
5648	dipeptidyl peptidase	226	1.2E-12	83.1%
7192	alpha-amylase precursor	207	2.9E-32	62.7%
7273	vanillyl-alcohol oxidase	146	5.3E-21	80.3%
10090	phospholipase d active site motif protein	235	3.3E-39	69.9%
10153	alpha-ketoglutarate dependent xanthine dioxygenase	249	1.2E-41	87.6%

### Prediction of secretory/signal peptides for the *S*. *sclerotiorum* contigs

A total of 2,754 coding regions were predicted with OrfPredictor from the set of 2,780 *S*. *sclerotiorum* ESTs. The peptide sequences were then used as a query for SignalP 3.0, which predicts the presence and location of signal peptide cleavage sites in amino acid sequences and identifies them as secretory proteins. The neural network (NN) method predicted 244 secretory signals, and the Hidden Markov Model (HMM) predicted 216. A total of 142 ESTs were identified by both NN and HMM and can be considered putative secretory peptides with high confidence (see Additional file 2). Of these 142 predicted secretory proteins, 21 were reported to be involved in pathogen virulence or pathogenicity (Table 6).

**Table 6:** ***S*. *sclerotiorum *EST contigs encoding potential secretory/signal peptides involved in virulence or pathogenicity**

**Seq**. **Name**	**Seq**. **Description**	**Seq.Length**	**min.e-value**	**mean Similarity**
355	Enolase	120	3.7E-14	97.8%
395	bzip transcription factor	108	2.5E-10	86.1%
1352	fkbp-type peptidyl-prolyl	479	2.4E-58	81.5%
1434	chitin synthase 1	220	2.6E-36	86.1%
2605	cysteine desulfurase	157	3.7E-14	93.8%
3499	vacuolar protein 8	255	7.7E-28	97.4%
3632	autophagy protein	253	2.8E-38	60.3%
4181	mfs toxin efflux pump	194	1.1E-21	69.4%
4467	nadh:ubiquinone oxidoreductase subunit	117	1.3E-16	90.8%
5493	formate nitrite transporter	145	9.4E-18	77.1%
6251	v-atpase proteolipid subunit	398	3.8E-43	89.4%
6759	endo-1,4-beta-xylanase	359	8.8E-45	81.1%
7392	rhamnogalacturonan acetylesterase	330	3.6E-38	73.3%
7736	phosphoethanolamine transferase pigf	186	7.7E-20	76.1%
8184	tetraspanin tsp3	225	2.5E-31	96.7%
8501	pectin methylesterase	252	1.6E-41	62.5%
9219	adenylate kinase	225	4.5E-12	100.0%
9240	glycosyl hydrolase family 61	320	1.5E-55	65.9%
9375	chd5 domain-containing protein	327	1.2E-41	82.2%
9461	phosphatidylglycerol phosphatidylinositol transfer protein	551	9.8E-92	70.5%
9847	Glutaredoxin	158	6.9E-21	83.9%

## Discussion

### Significance of study and summary of the main findings

Despite *Pisum sativum* being used by Gregor Mendel to propose a model of particulate inheritance and being a highly nutritious food source for populations worldwide, few genomic resources exist for pea. One of the pathogens of pea, *S*. *sclerotiorum* is not only capable of causing devastating disease of pea but is able to infect over 400 plant species [1]. By sequencing a normalized cDNA pool of the pea-*S*. *sclerotiorum* interaction with next generation sequencing we have catalogued a number of novel genes putatively involved in pathogenicity and resistance. To our knowledge this is the first study to examine the pea-*S*. *sclerotiorum* “interactome”. Sequencing the transcriptome (RNA-seq) is the method of choice in non-model systems for transcript discovery and genome annotation [8]. However, it has rarely been used to study plant-fungal interactions; one reason for this is the difficulty in distinguishing plant and fungal ESTs, particularly when reference genomes are not available. Using genomes of closely related species and tBLASTx to parse pea and *S*. *sclerotiorum* ESTs we demonstrated that Roche 454-pyrosequencing is a useful technique to characterize the host-pathogen interactome when genome resources are limited.

### tBLASTx parsing method

Two different strategies have been utilized previously to identify transcript origins in mixed plant and fungal EST datasets. One is a predictive method based on triplet nucleotide usage frequencies [9] and the other is a homology method using the BLASTp algorithm [10]. One shortcoming of the BLASTp method is that it could not be applied to novel genes or sequences from the non-coding regions of genes. Although the triplet nucleotide frequency method extends the application of the algorithm to both coding and non-coding sequences, the classification accuracy is approximately 90%, and required the use of a training set of ESTs to develop the nucleotide frequency for separation. A combined method was also used by Fernandez et al. [11], although this method distinguished 91% of the ESTs from the *Coffea arabicaHemileia vastatrix* interaction no validation of the method was presented [11].

Classification of genes from a pool of mixed cDNA by traditional sequence similarity analysis (BLAST) is of interest to many investigations into plant-pathogen interactions. DNA sequencing is becoming more affordable and whole genome sequences of many organisms are becoming available and will aid in plant-pathogen interaction studies. However, in pea these resources are not available, therefore, we used a standalone BLAST approach against proxy-reference genome databases with high genetic similarity to pea or *S*. *sclerotiorum* to distinguish mixed transcripts. Using an artificial mixture of known pea and *Sclerotinia* ESTs, we found the error rate using the BLAST method was significantly lower than the triplet nucleotide frequencies method (Table 7). We also demonstrated that the tBLASTx algorithm provided improved sorting of contigs relative to the BLASTn algorithm, and results in fewer ambiguous reads (see Additional file 3). In addition, although one individual genome of *S*. *sclerotiorum* (strain 1980) has been sequenced [12], there are still 1.6 Mb of predicted gaps in the 39.6 Mb assembly. To avoid ignoring unique genes between two different strains of the same species, a multi-fungal genome approach was adopted in this study. It was demonstrated that the assignment error rate based on 7 closely related fungal genomes was slightly decreased relative to assignment based on the single *S*. *sclerotiorum* genome (see Additional file 4). The e-value and e-value ratio utilized in our study to differentiate pea and *S*. *sclerotiorum* reads chosen selected after comparing several e-values, to maximize discrimination while reducing the error rate (see Additional file 5). Additionally, we determined error rates for this method using the artificial EST mix and validated the technique using our EST data set. We found that the percentage of unassigned ESTs (23.9%) in the 454 data set was higher than in the test EST data set (9%). One hypothesis to explain this difference is the average sequence length in the 454 data (200 bp) was shorter than known pea (496 bp) or *Sclerotinia* ESTs (674 bp) used in test data, which may result in no significant alignment against the proxy-reference plant or fungal genome databases, particularly when non-coding mRNA is considered. The remaining unassigned EST contigs (21%) were parsed using BLASTn searches against known pea and *S*. *sclerotiorum* ESTs, which assigned 44.4% of the unassigned contigs. Using this combined tBLASTx and BLASTn approach 89.4% of the 10,158 contigs were identified as pea (6,299) or *S*. *sclerotiorum* EST (2,780). Additionally, the EST parsing method was validated by PCR demonstrating that the parsing method was able to correctly assign ESTs from the 454 data set with a low error rate.

**Table 7:** **Comparison of triplet nucleotide frequencies method and tBlastx method to assign artificial EST mixture of pea-*S*. *sclerotiorum *(including 18,490 pea ESTs and 17,198** ***S*. *sclerotiorum *ESTs)**

**Category of EST**	**Number of ESTs by triplet nucleotide frequencies method**	**Number of ESTs by tBLASTx method**
Plant	17,660 (*2*,*180 wrong*)	15,289 (*14 wrong*)
Fungi	18,028 (*3*,*010 wrong*)	16,908 (*23 wrong*)
Ambiguous	0	1,519
Unassigned	0	1,972
Total	35,688	35,688

### Pea ESTs unique to the pea-*S*. *sclerotiorum* interaction

In response to pathogen attack, plants have evolved complex signaling and defense pathways. Putatively unique ESTs in our pea-*S*. *sclerotiorum* interactome were defined and identified by comparing EST contigs in our library against those of non-interaction EST libraries of pea and *S*. *sclerotiorum*. Although we identified a total of 2,840 (45.1%) putatively unique pea ESTs it was only possible to annotate 1,631 of these and only 451 had annotations suggesting roles in defense or response to biotic and abiotic stress. Most of the annotated genes are consistent with previous expression profiling analyses in *Brassica napus* infected with *Sclerotinia sclerotiorum*[13]. Following infection, many genes, including those encoding defense-associated proteins, enzymes involved in signaling pathways, and genes encoding transcription factors were induced.

Transcriptional control of the expression of stress-responsive genes is a crucial part of plant response to a range of abiotic and biotic stresses [14]. We demonstrated that 67 putative transcription factors were detected. These genes were classified into the MYB family, the Apetala2/Ethylene responsive element binding protein (AP2/EREBP) family, WRKY family and others (Table 2). Seven MYB family transcription factors were detected in our data and they play a key role in hormone signal transduction and disease resistance [15]. Eight AP2/EREBP transcription factors, including 3 ethylene insensitive transcription factors (contig 1311, 7671 and 8261) and 3 AP2/ERF genes (7472, 8894 and 9486), are key regulatory elements for ethylene signaling and response for biotic or abiotic stresses [16,17].WRKY40 act as negative regulators of defense signaling and have been associated with negatively regulating resistance to *P*. *syringae* in Arabidopsis [18].

Plant defenses are regulated through a complex network of transduction pathways [19]. Sixty-nine unique pea ESTs involved in signaling pathways were detected in this study. The signaling pathways were mediated by different signaling molecules, like abscisic acid (ABA), auxin, brassinosteroid, calcium ion, ethylene (ET), gibberellic acid (GA), jasmonic acid (JA), salicylic acid (SA) and small GTPase (Table 3). Those results were consistent with previous studies of signaling pathways involved in plant resistance to *Sclerotinia sclerotiorum*[20,21].

Expression of downstream proteins, including defense-associated proteins, was induced through signal transduction and transcription factor regulation after pathogen infection. In this study, 82 unique pea ESTs encoding defense related proteins were detected (Table 4). Four contigs (5422, 6766, 7235 and 9781), encoding putative pathogenesis-related (PR) proteins involved in the response to pathogen attack were prominent. Numbers of cell-wall-related genes were also detected; those contigs involved in the biosynthesis of plant cell wall structures and the disassembly of fungal cell walls. Chitinase (encoding by contig 491, 589, 1622, 2311 and 5742), beta-1, 3-glucanase (encoding by 196, 1243 and 4388) and other glycoside hydrolases are known to possess anti-fungal activity by degrading fungal cell walls [22].

### *S*. *sclerotiorum* ESTs unique to the pea-*S*. *sclerotiorum* interaction

Pathogens have evolved a number of strategies to gain entry into the host cell and to overcome the plant defense system. In this study, we identified 996 *S*. *sclerotiorum* contigs as specifically expressed during pea-*S*. *sclerotiorum* interaction through comparison of EST contigs against *S*. *sclerotiorum* ESTs from growth libraries. Ninety-five of 438 annotated contigs were described as being involved in pathogen virulence or pathogenicity (Table 5).

Fungi produce enzymes that degrade the cell wall and wall-associated polymers to penetrate plant cells. There were 39 specifically expressed contigs involved in the penetration of the plant cuticle and cell wall. Contig 6412 encodes an exoglucanase 2 precursor, which has cellulolytic activity [23] and is involved in cellulose degradation; enzymes encoded by 11 contigs (473, 555, 717, 970, 3115, 3513, 5562, 5918, 6759, 6383 and 9655) are involved in hemicellulose degradation; enzymes encoded by 11 contigs (1533, 3147, 3861, 5679, 6431, 6824, 7085, 7661, 7834, 8501 and 10069) are involved with pectin degradation. In addition, carbohydrate esterase encoded by contig 1562 was also involved in plant polysaccharide degradation. Integrity of the fungal cell wall is also very important for pathogenesis and some reports showed the deletion of biosynthetic cell wall enzymes resulted in dramatically reduced virulence [24]. In our data, 18 contigs were identified as affecting biosynthesis and integrity of fungal cell walls. Enzymes encoded by contigs 6654, 6797, 8474 and 8681 were involved in chitin synthesis; contigs 2424, 3362, 5737, 6956, 7397 and 9176 were involved in glucan synthesis; and 8 contigs (239, 330, 2930, 3825, 4843, 5773, 8785 and 9570) were involved in mannan synthesis.

*Sclerotinia sclerotiorum* differentiates appressoria into infection cushions prior to invasion and we found 12 genes involved in the formation of infection structures. Eight contigs were involved in response to the host immune system, of which 3 efflux transporters encoded by contigs 1220, 4783 and 6180 are responsible not only for export of compounds involved in pathogenesis such as secondary metabolites, but also export of host-derived antimicrobial compounds [25-27]. Contig 1769 had similarity to the guanine nucleotide-binding protein (G protein) alpha subunit which is an important signal transducing molecule in cells, essential for growth, asexual and sexual development, and virulence in both animal and plant pathogenic filamentous fungal species [28]. Importin beta-2 encoded by contig 3623 belongs to the importin β family which mediates transport between the nucleus and cytoplasm of macromolecules that contain nuclear import or export signals. All importin β members have the ability to recognize and bind specific cargo involved in the recognition of the host and signaling [29,30].

### Secreted/signaling proteins

Proteins secreted by fungi play a key role in the development of plant disease and the evolution of pathogenicity [31]. Some secreted proteins can degrade polymers encountered, such as cellulose, lipid, protein, and lignin, and transport the resulting simple sugars, amino acids, and fatty acids into the growing cell for use [32]. Using the SignalP3.0 program with stringent criteria, 142 contigs encoding putative secreted proteins were identified in the 2,780 *S*. *sclerotiorum* contigs. Twenty-one of the 66 annotated contigs were described as involved in pathogen virulence/pathogenicity in previous research (Table 6). Contig 355 encodes an enolase which is usually present on the cell surface or even secreted and is a potential virulence factor. In bacterial systems enolase has been demonstrated to contribute to pathogenicity by binding plasminogen in the infected host, potentially allowing the bacteria to acquire surface-associated proteolytic activity [33-35]. The basic leucine zipper transcription factor, encoded by contig 395, is a member of the bZIP family, one bZIP family member (Moatf1) from the rice fungus *Magnaporthe oryzae* mediates oxidative stress responses and is necessary for full virulence [36]. Contig 1352 encoding fkbp-type peptidylprolyl isomerase, with high homology to the Mip (macro-phage infectivity potentiator) protein, has been shown to be an essential virulence factor in *Legionella pneumophila*[37-39]. Chitin synthase 1 (contig 1434) plays a major role in cell wall biogenesis. Disruption of *Botrytis cinerea* class I chitin synthase gene *Bcchs1* results in cell wall weakening and reduced virulence [40,41]. Autophagy is necessary for turnover of organic matter during the formation of conidia and appressoria and for normal development and pathogenicity in *Magnaporthe grisea*. Autophagy is required for the virulence of some eukaryotic pathogens [42-44]. Contig 6759 encodes endo-β-1,4 xylanase which plays a significant role in the virulence of *Magnaporthe oryzae*, affects both penetration and expansion of *M*. *oryzae* in infected plants [45]. Pectin methylesterase (PME) produced by phytopathogenic bacteria and fungi catalyses the demethoxylation of pectin, a major plant cell wall polysaccharide [46]. The possible role of secreted adenylate kinase (AK), encoded by contig 9219, as a virulence factor is in producing and keeping an intact pool of toxic mixtures of AMP, ADP, and ATP, which allows *Pseudomonas aeruginosa* to exert its full virulence [47]. Glutathione reductase is important to nitric oxide and macrophage resistance and is essential for virulence [48] and in *Candida albicans* GRX2, a putative glutaredoxin, is required for virulence in a murine model [49].

## Conclusions

Here we present an EST resource that is specific for the pea-*S*. *sclerotiorum* interaction. We demonstrate and validate a method to reliably parse host and pathogen ESTs without the need for reference genomes. The ESTs were compared to non-interaction EST libraries to identify candidate resistance and pathogenicity genes. We also catalogued 145 proteins putatively secreted by *S*. *sclerotiorum*. The EST dataset will be a useful reference for further plant-fungus interaction studies, particularly for the *Sclerotinia* and legume research communities. Additionally, the *S*. *sclerotiorum* ESTs will be a valuable resource for the annotation of the *S*. *sclerotiorum* genome. Although the depth of our sequencing was not sufficient to obtain a global view of transcripts expressed during the pea-*S*. *sclerotiorum* interaction, the results are still very useful for the identification of plant resistance, fungal pathogenicity and virulence genes. This study sets the ground work and will be a resource for our current pea-*S*. *sclerotiorum* RNAseq expression profiling studies.

## Methods

### Plant, fungal growth and inoculation

Three plants of pea cultivar ‘Lifter’ (PI628276) were established per 1 gallon plastic pot in Sunshine LA 4 potting mix (Sun Gro Horticulture, Bellevue, WA). The plants were maintained in a greenhouse for 4 weeks with supplemental lighting extending the day length to approximately 14 h (October). Day and night temperatures were 22 ± 2°C and 16 ± 2°C, respectively. *S*. *sclerotiorum* isolate WMA-1 was isolated from a diseased pea plant in 2003 from a pea field (Washington, USA) with white mold disease symptoms and stored as air dry sclerotia at room temperature. Isolate WMA-1 (=ATCC MYA-4521) was demonstrated to be genetically representative of eight *S*. *sclerotiorum* strains sampled from legume hosts from various geographic locations using randomly amplified polymorphic DNA (RAPD) analysis (Kawabe and Peever, *unpublished*). Plants were inoculated with a 5 mm plug collected from the leading edge of an actively growing colony on a potato dextrose agar (PDA). The plug was placed fungal side down on the stem between the 4^th^ and 5^th^ detectable nodes and held in place by wrapping with Parafilm. Plants were transferred to a growth chamber with a 12 h photoperiod, an approximate 60% relative humidity, temperature of 20 ± 1°C and a 12 h photoperiod, for 72 hours to allow disease lesion development prior to RNA extraction.

### Total RNA extraction and purification of mRNA from total RNA

A 1 cm stem section was collected from each of 18 infected plants by cutting above and below the lesion front advancing toward the base of the plant. The stem section included both necrotic and green tissue with the advancing lesion front located in the center of the section. Stem sections were snap-frozen in liquid nitrogen and ground to a fine powder with a mortar and pestle. A total of 3 ml of TRIzol (Invitrogen, Carlsbad, CA, USA) was added to the ground tissue and the sample was split in half for column purification with the TRIzol Plus RNA purification kit (Invitrogen, Carlsbad, CA, USA). The additional step of on-column DNA digestion was performed with DNase I (Invitrogen, Carlsbad, CA, USA) to remove contaminating DNA. RNA was eluted in 250 μl of water per spin column. Poly-A RNA was isolated from total RNA with the Oligotex kit using the mRNA spin-column protocol (Qiagen, Valencia, CA, USA). Purified mRNA was eluted in a total of 100 μl of 5 mM Tris (pH 7.5). RNA and mRNA quantity was determined with a spectrophotometer (NanoDrop Technologies Inc., Wilmington, DE, USA). Total RNA and mRNA quality was assessed with an RNA Nano LabChip on an Agilent 2100 Bioanalyzer (Agilent Technologies, Santa Clara, CA, USA).

### cDNA synthesis, normalization and 454 pyrosequencing

Purified mRNA was used to construct a full length normalized cDNA pool through the services of Evrogen [50]. Briefly, the service utilized the SMART cDNA cloning methodology to generate a full length cDNA pool [51], which was normalized using a duplex-specific nuclease [52]. The double stranded normalized cDNA pool was sheared by nebulization and prepared for and sequenced as per manufacturer’s instructions on a Roche 454 GS FLX sequencer using an entire plate at Washington State University.

### Data filtering and *de novo* assembly

35 Mb of sequence data representing 162,729 reads were generated by 454 sequencing. Quality trimming, adaptor sequence removal and size selection of reads was performed with Galaxy software (http://main.g2.bx.psu.edu/) [53]. After trimming adaptors, 128,720 reads with quality scores over 20 and sequence length longer than 50 bp were assembled with Abyss [54]. Parameters were adjusted for optimal assembly as measured by N50 statistic (a weighted median statistic such that 50% of the entire assembly is contained in contigs or scaffolds equal to or larger than this value).

### Virus or viroid contamination detection

To determine whether any viruses or viroids were present in the fungi-infected plant cDNA sample, viroid and virus databases [55], including 41 complete viroid genomes and 2628 virus genomes, were downloaded from NCBI (released in April 2011). All EST contigs were analyzed with tBLASTx against viroid and virus databases. The e-value cutoff threshold was set at 1e^-3^. Contigs with a BLAST hit to viroid and virus databases were further analyzed by tBLASTx program against 3 legume genomes database and 7 fungi genomes database individually using the same cutoff threshold (see next section “development of a *S*. *sclerotiorum* and *P*. *sativum* parsing method”).

### Development of a *S*. *sclerotiorum* and *P*. *sativum* parsing method

To separate *S*. *sclerotiorum* and pea ESTs from the mixed pool, a procedure based on that proposed by Hsiang et al. in 2003 [56] was employed with modifications (Figure 2). Briefly, the mixed ESTs were compared with tBLASTx (NCBI-BLAST-2.2.24+) to fungal and plant “proxy-reference” genome databases (Table 8). These proxy reference databases were established as the pea genome is not available and the inclusion of additional ascomycetes genomes to *S*. *sclerotiorum* (strain 1980) improved the assignment rate. The proxy-fungal genome database was a mixture of *Sclerotinia sclerotiorum* (strain 1980) and 6 closely related Ascomycete fungi (*Botrytis cinerea*, *Chaetomium globosum*, *Fusarium graminearum*, *Magnaporthe grisea*, *Neurospora crassa and Verticillium dahlia*) and a plant genome database including 3 sequenced legume genomes (*Glycine max*, *Lotus japonicus and Medicago truncatula*). ESTs that only matched to fungal or plant genome database with an e-value of 1e^-03^ or better were automatically classified into *S*. *sclerotiorum* or pea ESTs, respectively. ESTs, which matched (e-value <1e^-3^) to both fungi and plant databases, were further analyzed by comparing the e-value of best-hit from fungi and plant genome results. An e-value ratio was determined by dividing the best-hit e-value to fungi and plant genomes from the tBLASTx searches. A cutoff ratio were set at > =1e^20^ for pea ESTs, <=1e^-20^ for *S*. *sclerotiorum* ESTs and those that fell between 1e^-20^ and <1e^20^ were considered to be ambiguous. To acquire a final sort of results, those ESTs without a BLAST hit or those found to be ambiguous were assigned with BLASTn against known *S*. *sclerotiorum* or pea ESTs if their identity was above 95% in similarity across 95% of the sequence length. 81,449 pea ESTs (from flowers, leaves, cotyledons, epi- and hypocotyl, and etiolated and light treated etiolated seedlings) [7] and 57,751 *S*. *sclerotiorum* ESTs (from mycelia growing at neutral pH, developing apothecia and developing sclerotia---downloaded from BROAD database) were used to assist in the classification and annotation of contigs.

**Table 8:** Source of fungal plant genome databases used for tBLASTx EST assignment

**7 ascomycete fungal genome databases**	**Size**	**Database**	**Version**
*Botrytis cinerea*	42.66 Mb	BROAD	2005-10
*Chaetomium globosum*	34.89 Mb	BROAD	2005-06
*Fusarium graminearum*	36.45 Mb	BROAD	2007-03
*Magnaporthe grisea*	38.76 Mb	BROAD	2011-04
*Neurospora crassa*	41.04 Mb	BROAD	2010-06
*Sclerotinia sclerotiorum*	38.33 Mb	BROAD	2011-06
*Verticillium dahlia*	33.83 Mb	BROAD	2008-07
**3 legume genome databases**	**Size**	**Database**	**Version**
*Glycine max*	~975 Mb	GenBank	2010-01
*Lotus japonicus*	~500 Mb	GenBank	2008-06
*Medicago truncatula*	~305 Mb	GenBank	2009-03

To verify the feasibility of the EST parsing method, 17,533 *S*. *sclerotiorum* ESTs derived from developing *S*. *sclerotiorum* libraries were downloaded from BROAD institute and 18,547 *P*. *sativum* ESTs were obtained from the GenBank EST database by search keyword ‘*Pisum sativum*’. Vector contamination was removed from the downloaded ESTs by BLAST search with UniVec database (GenBank) in *P*. *sativum* and *S*. *sclerotiorum* ESTs were trimmed. After vector trimming, tBLASTx analysis of the downloaded ESTs was performed separately against the proxy-reference fungal and plant databases (Table 8). The following relevant data from tBLASTx output were extracted to an Excel file: query sequence name, query sequence length, fungi database target name, fungi database e-value for top match, total query sequence length for all match to fungi database, plant database target name, plant database top match e-value, total query sequence length for all match to plant database.

### PCR to confirm validity of classified contigs

Fifty contigs from *S*. *sclerotiorum* and 50 contigs from pea were randomly sampled to check the validity of EST contig classification. Primers were designed for each contig using the program Primer3 [57]. cDNA from pea inoculated with *S*. *sclerotiorum*, cDNA from non-inoculated pea, cDNA from *S*. *sclerotiorum* growing on PDA medium, and genomic DNA extracted from pea and *S*. *sclerotiorum* using DNeasy plant mini kit (Qiagen, Valencia, CA, USA ) were used as template in PCR with primer pairs for each contig. PCR contained 4 μl of 5 × GoTaq PCR Buffer (Promega, Madison, WI, USA), 200 μM each dNTP, 2.5 μM each primer, 0.4 U of GoTaq polymerase, and approximately 50 ng of DNA template in a final volume of 20 μl. PCR were held at 94°C for 2 min; followed by 40 cycles of 94°C for 30 s, 60°C for 30 s, and 72°C for 1 min; with a final extension at 72°C for 10 min. PCR products from each contig were separated on a 1% agarose gel and visualized with ethidium bromide.

### Gene annotation and analysis

The biological function of EST contigs was predicted with gene ontology (GO) terms based on BLASTx analysis using the program BLAST2GO [58,59]. Default BLASTx parameters with an e-value threshold of 1e^-3^ and a high-scoring segment pairs (hsp) filter of 33 were retained so as to assign function to as many contigs as possible while ensuring short matching sequences less than 100 nucleotides were excluded. An annotation configuration with e-value-hit-filter 1.0E^-6^, annotation cut off “55” and GO weight “10” was selected.

### Prediction of secretory/signal peptides for the *S*. *sclerotiorum* ESTs

The secretory/signal peptides for each *S*. *sclerotiorum* EST were analyzed using prediction algorithms. Firstly, OrfPredictor [60] was used to predict protein coding regions for the assembled ESTs. The output for OrfPredictor was a file of predicted coding regions from the ESTs in FASTA format, where the definition line contains the query identifier, the frame, the beginning and the end position of the predicted coding region, and the predicted protein peptide sequences. The peptide sequences were then used as a query for SignalP 3.0 (http://www.cbs.dtu.dk/services/SignalP/) with default settings, which predicts the presence and location of signal peptide cleavage sites [13]. Both a neural network (NN) and Hidden Markov Model (HMM) approach were used. EST contigs identified by both NN and HMM were considered to be secretory/signal peptides with high confidence.

## Competing interests

The authors declare that they have no competing interest.

## Authors’ contributions

XF performed bioinformatics analysis, validated the EST parsing and drafted the manuscript. KM provided pea lines for analysis and contributed to direction of the study. TC participated in design of the study and conducted signal peptide analysis. TP initiated the project and directed the study. MC directed the project, performed inoculations and initial experiments to develop the normalized cDNA and drafted the manuscript. All authors read, edited and approved the final manuscript.

## Authors’ information

XF is a Research Associate at Michigan State University with a background and interest in expression profiling. KM is an Associate Professor and legume breeder in the Department of Plant Sciences at North Dakota State University. TC is Trait Production Manager at Dow AgroSciences LLC. TP is an Associate Professor of Plant Pathology at Washington State University, with particular interests in the mechanisms of fungal speciation and the genetics of host specificity. MC is an Assistant Professor of Plant Pathology at Michigan State University.

## Supplementary Material

Additional file 1 **Fasta files for 10158 contigs (pea and Sclerotinia ESTs), parsed into ambiguous contigs (1a), no blast-hit contigs (1b), pea contigs (1c) and*S. sclerotiorum*contigs (1d).** Click here for file

Additional file 2 ***S. sclerotiorum*EST contigs encoding potential secretory/signal peptides.** Click here for file

Additional file 3 **Comparison of BLASTn and tBLASTx method to assign artificial EST mixture of pea-*S. sclerotiorum*(including 18,490 pea ESTs and 17,198 *S. sclerotiorum*ESTs).** Click here for file

Additional file 4 **The assignment results of an artificial EST mixture using the tBlastx method against 7 fungal genome databases and the*S. sclerotiorum*genome only.** Click here for file

Additional file 5 Comparison of different e-value ratios (fungi/plant) to distinguish species ESTs from the artificial EST mixture. Click here for file
