# Supplementary material for: Rapid transcriptome characterization and parsing of sequences in a non-model host-pathogen interaction; pea-Sclerotinia sclerotiorum
Source: BMC Genomics. 2012 Nov 26;13:668. doi: 10.1186/1471-2164-13-668 (PMC3534286; doi:10.1186/1471-2164-13-668)
Supplement: Additional file 2 — S. sclerotiorumEST contigs encoding potential secretory/signal peptides. [file 1471-2164-13-668-S2.docx]

# Additional file 2 –*S. sclerotiorum* EST contigs encoding potential secretory/signal peptides.

| **Seq. Name** | **Seq. Description** | **Seq. Length** | **min. e-value** | **mean Similarity** |
| --- | --- | --- | --- | --- |
| **Genes involved in virulence or pathogenicity of pathogen** | | | | |
| 355 | Enolase | 120 | 3.7E-14 | 97.8% |
| 395 | bzip transcription factor | 108 | 2.5E-10 | 86.1% |
| 1352 | fkbp-type peptidyl-prolyl | 479 | 2.4E-58 | 81.5% |
| 1434 | chitin synthase 1 | 220 | 2.6E-36 | 86.1% |
| 2605 | cysteine desulfurase | 157 | 3.7E-14 | 93.8% |
| 3499 | vacuolar protein 8 | 255 | 7.7E-28 | 97.4% |
| 3632 | autophagy protein | 253 | 2.8E-38 | 60.3% |
| 4181 | mfs toxin efflux pump | 194 | 1.1E-21 | 69.4% |
| 4467 | nadh:ubiquinone oxidoreductase subunit | 117 | 1.3E-16 | 90.8% |
| 5493 | formate nitrite transporter | 145 | 9.4E-18 | 77.1% |
| 6251 | v-atpase proteolipid subunit | 398 | 3.8E-43 | 89.4% |
| 6759 | endo-1,4-beta-xylanase | 359 | 8.8E-45 | 81.1% |
| 7392 | rhamnogalacturonan acetylesterase | 330 | 3.6E-38 | 73.3% |
| 7736 | phosphoethanolamine transferase pigf | 186 | 7.7E-20 | 76.1% |
| 8184 | tetraspanin tsp3 | 225 | 2.5E-31 | 96.7% |
| 8501 | pectin methylesterase | 252 | 1.6E-41 | 62.5% |
| 9219 | adenylate kinase | 225 | 4.5E-12 | 100.0% |
| 9240 | glycosyl hydrolase family 61 | 320 | 1.5E-55 | 65.9% |
| 9375 | chd5 domain-containing protein | 327 | 1.2E-41 | 82.2% |
| 9461 | phosphatidylglycerol phosphatidylinositol transfer protein | 551 | 9.8E-92 | 70.5% |
| 9847 | Glutaredoxin | 158 | 6.9E-21 | 83.9% |
| **Others** | | | | |
| 52 | N/A | 252 |  |  |
| 240 | N/A | 159 |  |  |
| 242 | hypothetical protein SS1G_10406 | 141 | 9.1E-18 | 89.0% |
| 292 | nascent polypeptide-associated complex subunit alpha | 194 | 6.9E-05 | 100.0% |
| 301 | lsm domain-containing protein | 243 | 4.7E-14 | 89.1% |
| 374 | uv-damaged dna-binding protein | 255 | 2.6E-44 | 65.2% |
| 579 | hypothetical protein SS1G_03913 | 207 | 4.9E-11 | 97.0% |
| 621 | N/A | 130 |  |  |
| 780 | N/A | 205 |  |  |
| 821 | plasma membrane proteolipid 3 | 360 | 1.0E-29 | 77.0% |
| 830 | hypothetical protein BC1G_15836 | 248 | 3.6E-22 | 66.0% |
| 832 | predicted protein | 244 | 9.2E-18 | 100.0% |
| 884 | N/A | 110 |  |  |
| 1052 | N/A | 141 |  |  |
| 1158 | N/A | 108 |  |  |
| 1204 | N/A | 242 |  |  |
| 1325 | pectin | 206 | 1.3E-11 | 100.0% |
| 1331 | hypothetical protein SS1G_01838 | 244 | 6.6E-16 | 93.5% |
| 1334 | N/A | 195 |  |  |
| 1475 | protein | 238 | 1.3E-19 | 81.6% |
| 1699 | N/A | 103 |  |  |
| 1722 | N/A | 278 |  |  |
| 1770 | N/A | 247 |  |  |
| 1772 | N/A | 167 |  |  |
| 1805 | phosphoribosylformylglycinamidine cyclo-ligase | 174 | 7.2E-23 | 74.3% |
| 1892 | duf1275 domain protein | 383 | 4.8E-59 | 72.0% |
| 1922 | N/A | 162 |  |  |
| 2147 | predicted protein | 143 | 3.7E-19 | 100.0% |
| 2150 | N/A | 122 |  |  |
| 2338 | N/A | 211 |  |  |
| 2393 | 60s ribosomal protein l15 | 196 | 7.3E-31 | 91.6% |
| 2413 | N/A | 106 |  |  |
| 2708 | predicted protein | 343 | 1.2E-49 | 60.4% |
| 2752 | hypothetical protein SS1G_03905 | 190 | 4.1E-24 | 95.5% |
| 2776 | N/A | 149 |  |  |
| 2931 | eukaryotic translation initiation factor 3 | 199 | 1.3E-30 | 100.0% |
| 3049 | hypothetical protein SS1G_11716 | 252 | 1.3E-14 | 98.5% |
| 3190 | N/A | 122 |  |  |
| 3307 | N/A | 114 |  |  |
| 3331 | integral membrane protein | 240 | 5.4E-26 | 70.7% |
| 3358 | pantothenate transporter liz1 | 243 | 1.2E-28 | 85.3% |
| 3545 | N/A | 101 |  |  |
| 3561 | hypothetical protein SS1G_00198 | 176 | 9.1E-26 | 77.8% |
| 3686 | hypothetical protein SS1G_00876 | 169 | 7.8E-25 | 77.1% |
| 3850 | N/A | 175 |  |  |
| 4045 | hit domain protein | 149 | 2.6E-20 | 77.8% |
| 4138 | duf887 domain-containing protein | 350 | 2.9E-51 | 84.9% |
| 4338 | predicted protein | 258 | 6.3E-22 | 90.0% |
| 4535 | N/A | 131 |  |  |
| 4640 | hypothetical protein SS1G_06834 | 236 | 2.9E-19 | 93.4% |
| 4685 | N/A | 146 |  |  |
| 4687 | N/A | 146 |  |  |
| 4700 | aryl-alcohol dehydrogenase | 107 | 3.8E-11 | 85.6% |
| 4743 | histone h2a | 320 | 7.6E-52 | 97.4% |
| 4786 | N/A | 130 |  |  |
| 4806 | hypothetical protein SS1G_10606 | 142 | 1.0E-08 | 100.0% |
| 4935 | hypothetical protein SS1G_12768 | 112 | 1.1E-13 | 100.0% |
| 5009 | 54s ribosomal protein l31 | 183 | 8.8E-16 | 82.2% |
| 5016 | N/A | 126 |  |  |
| 5064 | N/A | 133 |  |  |
| 5115 | dolichyl-phosphate mannosyltransferase polypeptide 3 | 201 | 1.5E-28 | 84.9% |
| 5117 | nitrogen regulatory protein otam | 224 | 6.1E-25 | 60.7% |
| 5264 | heat shock protein 30 | 134 | 8.6E-16 | 86.7% |
| 5269 | N/A | 331 |  |  |
| 5277 | kynurenine 3-monooxygenase | 117 | 9.6E-15 | 82.6% |
| 5332 | er membrane duf1077 domain-containing protein | 199 | 6.4E-27 | 80.5% |
| 5617 | 40s ribosomal protein s15 | 355 | 8.9E-37 | 95.8% |
| 5817 | hypothetical protein SS1G_04171 | 186 | 1.2E-12 | 98.0% |
| 5928 | proteasome regulatory particle subunit | 177 | 7.7E-17 | 98.7% |
| 5985 | hypothetical protein BC1G_04414 | 109 | 5.2E-08 | 97.0% |
| 5993 | hypothetical protein SS1G_11879 | 138 | 6.3E-19 | 96.5% |
| 6001 | N/A | 269 |  |  |
| 6038 | 60s ribosomal protein l31 | 120 | 1.3E-14 | 99.9% |
| 6126 | N/A | 212 |  |  |
| 6203 | hypothetical protein SS1G_13918 | 191 | 9.0E-29 | 99.0% |
| 6359 | ell complex subunit | 164 | 3.3E-23 | 85.9% |
| 6455 | N/A | 131 |  |  |
| 6570 | N/A | 102 |  |  |
| 6589 | hypothetical protein SS1G_13386 | 189 | 3.0E-16 | 95.0% |
| 7033 | protein | 152 | 1.1E-18 | 97.0% |
| 7092 | protein sys1 | 181 | 4.4E-20 | 89.1% |
| 7211 | opsin 1 | 152 | 1.5E-20 | 79.1% |
| 7225 | N/A | 233 |  |  |
| 7285 | hypothetical protein SS1G_07251 | 175 | 4.7E-06 | 100.0% |
| 7287 | N/A | 155 |  |  |
| 7332 | N/A | 185 |  |  |
| 7358 | N/A | 246 |  |  |
| 7439 | N/A | 123 |  |  |
| 7532 | hypothetical protein SS1G_13764 | 406 | 5.5E-71 | 61.0% |
| 7669 | hypothetical protein SS1G_05782 | 309 | 1.8E-18 | 100.0% |
| 7734 | 40s ribosomal protein s20 | 218 | 4.2E-15 | 96.9% |
| 7777 | N/A | 102 |  |  |
| 8167 | plasma membrane proteolipid 3 | 246 | 4.0E-29 | 87.8% |
| 8258 | biotin synthase | 256 | 7.9E-41 | 72.0% |
| 8286 | N/A | 103 |  |  |
| 8446 | N/A | 195 |  |  |
| 8479 | predicted protein | 128 | 3.0E-16 | 87.5% |
| 8559 | N/A | 204 |  |  |
| 8580 | mitochondrial carrier protein yhm1 | 150 | 2.8E-22 | 91.4% |
| 8813 | N/A | 145 |  |  |
| 8955 | N/A | 153 |  |  |
| 8981 | hypothetical protein SS1G_13636 | 269 | 5.6E-47 | 63.4% |
| 9020 | 60s ribosomal protein l29 | 112 | 5.3E-13 | 98.5% |
| 9116 | maltose permease | 255 | 2.0E-36 | 66.3% |
| 9117 | 60s ribosomal protein l5 | 241 | 2.5E-39 | 88.5% |
| 9138 | elongation factor 3 | 108 | 7.7E-12 | 81.8% |
| 9226 | N/A | 116 |  |  |
| 9347 | N/A | 230 |  |  |
|  |  |  |  |  |
| 9387 | isocitrate dehydrogenase subunit 1 | 129 | 1.2E-17 | 100.0% |
| 9420 | short chain dehydrogenase reductase family | 243 | 1.2E-28 | 85.4% |
| 9467 | glycerol kinase | 212 | 7.2E-31 | 78.1% |
| 9516 | N/A | 287 |  |  |
| 9532 | N/A | 244 |  |  |
| 9576 | nuclear migration protein nudf | 180 | 7.9E-22 | 70.5% |
| 9815 | hypothetical protein SS1G_03289 | 144 | 1.7E-22 | 94.5% |
| 9846 | nascent polypeptide-associated complex subunit alpha | 208 | 7.8E-17 | 95.2% |
| 9924 | 40s ribosomal protein s4 | 436 | 3.7E-71 | 92.1% |
| 9946 | mitochondrial import protein | 448 | 5.6E-39 | 80.2% |
| 9967 | N/A | 212 |  |  |
| 10117 | vacuolar membrane pq loop repeat protein | 236 | 3.1E-37 | 76.4% |
| 10134 | proteasome subunit beta type 7 precursor | 194 | 1.3E-14 | 97.5% |
