# Supplementary material for: Rapid transcriptome characterization and parsing of sequences in a non-model host-pathogen interaction; pea-Sclerotinia sclerotiorum
Source: BMC Genomics. 2012 Nov 26;13:668. doi: 10.1186/1471-2164-13-668 (PMC3534286; doi:10.1186/1471-2164-13-668)
Supplement: Additional file 3 — Comparison of BLASTn and tBLASTx method to assign artificial EST mixture of pea-S. sclerotiorum(including 18,490 pea ESTs and 17,198 S. sclerotiorumESTs). [file 1471-2164-13-668-S3.docx]

**Additional file 3 – Comparison of BLASTn and tBLASTx method to assign artificial EST mixture of pea-*S. sclerotiorum* (including 18,490 pea ESTs and 17,198 *S. sclerotiorum* ESTs).**

| **Category of EST** | **Number of ESTs by BLASTn method** | **Number of ESTs by tBLASTx method** |  |
| --- | --- | --- | --- |
| Plant | 11,724 | 15,289 *(14 wrong)* |  |
| Fungi | 16,803 | 16,908 *(23 wrong)* |  |
| Ambiguous | 217 | 1,519 |  |
| Unassigned | 6,944 | 1,972 |  |
| **Total** | 35,688 | 35,688 |  |
