# Supplementary material for: Rapid transcriptome characterization and parsing of sequences in a non-model host-pathogen interaction; pea-Sclerotinia sclerotiorum
Source: BMC Genomics. 2012 Nov 26;13:668. doi: 10.1186/1471-2164-13-668 (PMC3534286; doi:10.1186/1471-2164-13-668)
Supplement: Additional file 4 — The assignment results of an artificial EST mixture using the tBlastx method against 7 fungal genome databases and theS. sclerotiorumgenome only. [file 1471-2164-13-668-S4.docx]

**Additional file 4 – The assignment results of an artificial EST mixture using the tBlastx method against 7 fungal genome databases and the *S. sclerotiorum* genome only.**

| **Category of EST** | **Number of ESTs by against *S. sclerotiorum* genome only** | **Number of ESTs by against 7 fungi genome databases** |  |
| --- | --- | --- | --- |
| Plant | 15,558 *(49 wrong)* | 15,289 *(14 wrong)* |  |
| Fungi | 16,651 *(18 wrong)* | 16,908 *(23 wrong)* |  |
| Ambiguous | 1,397 (3.9%) | 1,519 (4.3%) |  |
| Unassigned | 2,082 (5.8%) | 1,972 (5.5%) |  |
| **Total** | **35,688** | **35,688** |  |
