# Supplementary material for: Rapid transcriptome characterization and parsing of sequences in a non-model host-pathogen interaction; pea-Sclerotinia sclerotiorum
Source: BMC Genomics. 2012 Nov 26;13:668. doi: 10.1186/1471-2164-13-668 (PMC3534286; doi:10.1186/1471-2164-13-668)
Supplement: Additional file 5 — Comparison of different e-value ratios (fungi/plant) to distinguish species ESTs from the artificial EST mixture. [file 1471-2164-13-668-S5.docx]

**Additional file 5 – Comparison of different e-value ratios (fungi/plant) to distinguish species ESTs from the artificial EST mixture.**

| **Category of EST** | **e-value ratios pick up by e^-10^ and e^10^** | **e-value ratios pick up by e^-20^ and e^20^** | **e-value ratios pick up by e^-30^ and e^30^** |
| --- | --- | --- | --- |
| Plant | 16,224 *(29 wrong)* | 15,289 *(14 wrong)* | 14,505 *(11 wrong)* |
| Fungi | 16,944 *(23 wrong)* | 16,908 *(23 wrong)* | 16,814 *(23 wrong)* |
| Ambiguous | 548 (1.55%) | 1,519 (4.3%) | 2,397 (6.7%) |
| Unassigned | 1,972 (5.5%) | 1,972 (5.5%) | 1,972 (5.5%) |
| **Total** | **35,688** | **35,688** | **35,688** |
